# Emotional intelligence among nurses in a general hospital setting in Jeddah, Saudi Arabia

**DOI:** 10.3389/fmed.2025.1610150

**Published:** 2025-06-24

**Authors:** Ibrahim Yahya Alhakami, Omar Ghazi Baker

**Affiliations:** 1 College of Nursing, King Saud University, Riyadh, Saudi Arabia; 2 Department of Community Psychiatric and Mental Nursing, College of Nursing, King Saud University, Riyadh, Saudi Arabia

**Keywords:** emotional intelligence, level, influencing factors, nursing, general hospitals, Saudi Arabia

## Abstract

**Aim:**

Emotional intelligence (EI) is a prerequisite of nursing practice. It significantly affects the wellbeing and quality of the provided services. The literature is scarce regarding the EI levels of Saudi Arabia’s nursing professionals. Therefore, this study aims to assess EI levels and explore influencing factors among nurses in a general hospital in Jeddah, Saudi Arabia.

**Methods:**

A cross-sectional design was used involving a convenience sample of 80 registered nurses, and using the Trait Emotional Intelligence Questionnaire Short Form (TEIQue SF) tool for the collection of data. Data were analyzed through ANOVA and independent sample *t*-test at a *p*-value of < 0.05.

**Results:**

The results indicated a moderate TEIQue global score (3.3 ± 0.33), with wellbeing scoring highest (3.7 ± 0.38), followed by sociability (3.3 ± 0.42). In constrast, self-control (3.2 ± 0.37) and emotionality (3.1 ± 0.41) received comparatively lower scores. Female nurses had significantly higher scores of wellbeing, and non-Saudi nurses presented high scores of wellbeing (3.7 ± 0.37) and emotionality (3.2 ± 0.46). Less work experience was associated with a higher global score (3.4 ± 0.40). The regression analysis further revealed a high global score (3.6 ± 0.35) among nurses in the obstetric department.

**Conclusion:**

Nurses demonstrated a moderate level of EI, with wellbeing receiving the highest score, followed by sociability. Gender, nationality, and department were identified as influencing factors on EI levels. These findings emphasize the importance of EI-specific training programs to improve self-control and emotionality. Further longitudinal studies are needed to explore the effects of EI interventions on the skills of nurses and the quality of care of patients.

## 1 Introduction

Emotional intelligence (EI) is crucial for effective patient care, communication, and stress management. A high EI score among nurses in high-pressure healthcare settings facilitates enhanced interpersonal difficulty management and empathetic care (1). The EI concept emerged from psychological studies on emotions, abilities, and thoughts in the 1990s and has expanded during the last 25 years (2). EI’s recent applications in nursing, management, education, and business demonstrate its broad relevance (1).

EI refers to “an individual’s ability to recognize and differentiate their own and others’ emotions to guide thoughts and actions.” It encompasses self-awareness, emotion regulation, relationship management, challenge tackling, delayed gratification, overcoming obstacles for sustained problem-solving, and being optimistic (2). EI significantly enhances teamwork, supportive work culture, and conflict resolution, particularly in the high-stress nursing profession (3). EI specifically promotes nurses’ self-awareness and management, social awareness, and relationship management by understanding others in various situations (4–7). Nurses’ higher EI leads to greater empathy, emotional control, and active listening for improved patient satisfaction with fewer medical errors and better compliance with treatment regimens (8).

Factors influencing EI among nurses include years of experience, gender, workplace environment, and access to training. Experienced nurses often develop higher EI through refined interpersonal skills, while supportive work environments and EI training enhance emotional regulation and empathy (9, 10). Conversely, high stress or limited training may hinder EI, negatively affecting care quality (10).

Petrides et al. described Trait EI as a combination of emotional tendencies and self-assessments present at the foundation of personality hierarchies, which can be evaluated using the Trait Emotional Intelligence Questionnaire (TEIQue) tool. The model highlights personal emotional experiences, such as adaptability, emotional control, and self-worth, as enduring traits instead of changeable skills. In the nursing profession, Trait EI reflects the influence of inherent emotional tendencies on wellbeing, resilience, and collaboration under stressful situations, while fulfilling the daily interpersonal demands (11, 12).

Previous EI-related studies primarily focused on nursing students and mental health nursing (13). There is a paucity of literature on EI among frontline nurses in general hospitals. The Saudi Arabian nursing sector operates within a rapidly progressing framework based on Vision 2030 (14). The revolutionary steps involve technological innovations, the Saudization of the nursing staff, and patient-centered care. These measures necessitate enhanced EI skills among nurses. Thus, EI investigation in this transitional period could facilitate decision-makers to devise novel strategies for improved EI among nurses.

Accordingly, the current study addresses the gap in knowledge regarding the EI levels and the factors influencing them among nurses in general hospitals. The study aims to inform EI training strategies to improve patient care and team dynamics. It is grounded in the Trait Emotional Intelligence Theory using the Trait Emotional Intelligence Questionnaire Short Form (TEIQue-SF) (11, 12). The study seeks to answer a specific question: What are the EI levels among nurses in a general hospital in Jeddah, Saudi Arabia, and what factors influence these levels? We hypothesize that nurses’ EI is significantly influenced by their sociodemographic and practical characteristics.

## 2 Methods

### 2.1 Study design and setting

The study aimed to assess EI and its influencing factors among nurses in a general hospital. A cross-sectional survey was used, appropriate for obtaining data at a single point in time and analyzing the association with various predictors (15). A qualitative design was considered but viewed as less appropriate for quantifying the EI levels using the validated TEIQue-SF tool.

The study was conducted in a 500-bed general hospital, with approximately 600 registered nurses comprising medical (≈30%), surgical (≈22%), obstetric (≈15%), pediatric (≈11%), and outpatient clinics (≈10%), reflecting typical Saudi healthcare settings. A sample of 80 nurses was selected using a convenient sampling approach to ensure representativeness of the sample. The sample was estimated using G*Power to have sufficient statistical power for regression analysis. The power analysis indicated the need for approximately 85 participants. The final sample of 80 nurses produced a post-hoc power of 0.78, which is appropriate for the identification of moderate effects and regression analysis with 8 independent predictors (16).

Potential participants were assigned according to the hospital’s nursing schedule. The recruitment procedure involved announcements at staff meetings and emails through nursing administration from June to August 2024. Data were collected face-to-face by trained researchers. Nurses’ inclusion was based on (1) registered nurses currently working at the hospital (2), a minimum of 6 months’ experience, and (3) readiness to participate. The exclusion criteria comprised (1) nurses with temporary contracts (2), individuals on leave during the study timeframe, and (3) unregistered nursing personnel (aides). In total, 80 out of 100 contacted nurses agreed to take part, yielding an 80% response rate. Twenty nurses declined the proposal because of time limitations (*n* = 15) and no interest (*n* = 5). Participants filled out a 30-item TEIQue-SF, which was estimated to take approximately 15–20 min based on a pilot test with five nurses. Ethical aspects included obtaining participants’ written informed consent, guaranteeing voluntary involvement with the option to withdraw, and ensuring confidentiality through unique identifiers instead of names. Participants were not offered any incentives to prevent coercion. Moreover, the participants were informed about the study’s aim of improving nursing practice without revealing specific hypotheses to reduce response bias. Pilot validation was applied on 10 nurses, which revealed the applicability of the study tools, with no necessary modifications, and their results were included in the main results. The study was ethically approved by the Institutional Research Board (IRB) in the Directorate of Health Affairs in Jeddah.

### 2.2 Tool of the study [trait emotional intelligence questionnaire short form (TEIQue-SF)]

TEIQue-SF was devised by Petrides and Furnham in 2006 to assess the EI of a person (12). Rasch analysis and confirmatory factor analysis confirmed the study tool’s validity and reliability (α = 0.86) (17). The questionnaire comprised 30 questions on a 5-point Likert scale for EI measurement. The statements were categorized under four domains of EI skills, including wellbeing, self-control, emotionality, and sociability. Wellbeing indicates the extent of an individual’s fulfillment and satisfaction in his/her life with high self-esteem. Self-control refers to the degree of an individual’s control over his/her desires. Emotionality demonstrates a wide array of emotion-related skills in a person, such as the recognition of internal emotions and the perception and expression of emotions with friends and family. The sociability domain evaluates the individual’s influence in various social contexts rather than only in personal relationships with friends and family. The scale used a 5-point response format as strongly agree = 5, agree = 4, neutral = 3, disagree = 2, and strongly disagree = 1. Participants filled out the questionnaire using the self-reporting method in the presence of the researcher, who helped the participants with any vague statements. This tool included nurses’ sociodemographic questions (residence, gender, age, educational level, and social status) along with job-related queries (position, nature of work, and experience). The internal consistency was assessed using Cronbach’s alpha to verify the tool’s reliability in the sample. TEIQue-SF presented strong reliability with a Cronbach’s alpha of 0.85 for the overall trait EI score. Thus, the tool demonstrated internal consistency among Saudi nurses and affirmed its appropriateness for EI assessment within this hospital setting. The summation of the individual item scores within each domain, followed by the division by the total number of items, specifically produced a score for that domain between 1 and 5. Similarly, the global TEIQue score was calculated by adding the scores of all items from various domains and dividing by the overall number of items, which guaranteed a final score range between 1 and 5. These scoring methods facilitated a uniform comparison across fields and ensured consistency in the evaluation system.

SPSS software (version 26) was used for the data analysis, whereas the Shapiro–Wilk test was performed to test the normality of the scores. Independent sample *t*-tests and one-way ANOVA differentiated EI levels among groups at a significance level of *p* < 0.05. The t-test differentiated EI scores between two-group variables, such as gender (male and female), whereas ANOVA demonstrated variations among multiple variables, such as education (diploma, bachelor’s, and master’s). These assessments revealed EI variations among group factors and depicted the correlation between demographic variables and EI levels. A multiple regression analysis evaluated the EI-influencing factors. The global TEIQue-SF score served as the dependent variable, whereas the independent variables included years of experience, education level (dummy-coded: 0 = diploma, 1 = bachelor’s, and 2 = master’s), and department. Regression analysis demonstrated the combined and individual impacts of these predictors on EI while addressing confounding. The residual plots and variance inflation factors (VIF < 5) verified the assumptions (normality and multicollinearity) and thus validated the model. Two out of 80 completed questionnaires (*n* = 2, 2.5%) contained incomplete answers. To address such instances, listwise deletion was performed in regression analysis, which reduced the effective sample size to 78 as the absence of data was minimal and appeared random (Little’s MCAR test, *p* = 0.62). Mean substitution was applied for the single missing items in t-tests and ANOVA to include all 80 participants for strong group comparisons. None of the participants were completely excluded as all fulfilled the inclusion criteria.

## 3 Results

This study aimed to assess EI levels and identify influencing factors (e.g., experience, gender, nationality, department, and education) among nurses in a general hospital. The characteristics of the study group (*n* = 80) are presented in Table 1. The participants had an almost equal distribution of Saudi Arabian citizens (53.8%) and non-Saudis (46.3%), with a significant dominance of female nurses (92.5%). Most respondents (68.8%) were aged between 30 and 39 years old, whereas the majority (71.2%) had at least a bachelor’s degree, and only 28.8% were diploma holders. Approximately two-thirds (61.2%) of the nurses were married. Participants were gathered from various hospital units, with the highest participation from General Medicine (27.5%), Surgery (22.5%), and Obstetrics (15.0%) departments. The experience in their current specialty was noted as <5 years (31.3%), 5 to <10 years (30.0%), and 10 or more years (38.7%). Thus, the percentage remained slightly higher for individuals over 10 years of experience. The participants’ experience in the current hospital was reported as <5 years (30.0%), 5 to 10 years (30.0%), and >10 years (40.0%).

**Table 1:** Sociodemographic and professional characteristics of the participants (*n* = 80).

Characteristics	*N*	%
Nationality		
Saudi	43	53.8%
Non-Saudi	37	46.3%
Sex		
Male	6	7.5%
Female	74	92.5%
Age categories		
<30 years	20	25.0%
30–39 years	55	68.8%
40 + years	5	6.3%
Mean ± SD	33.4 ± 6.40	
Qualification		
Diploma	23	28.8%
Bachelor and postgraduate	57	71.2%
Marital status		
Single	31	38.8%
Married	49	61.2%
Qualification		
Diploma	31	38.8%
Bachelor	48	60.0%
Post-graduate	1	1.3%
Department/unit		
General medicine	22	27.5%
Surgery	18	22.5%
Obstetrics	12	15.0%
Pediatrics	9	11.3%
OPD	8	10.0%
Endoscopy	11	13.8%
Years of experience in the current specialty		
<5 years	25	31.3%
5–<10 years	24	30.0%
10 + years	31	38.7%
Years of experience in the current hospital		
<5 years	24	30.0%
5–<10 years	24	30.0%
10 + years	32	40.0%

SD, Standard deviation; OPD, outpatient department.

Table 2 presents the average EI scores at self-reported levels in different domains of participants. The table also demonstrates moderate and proportionate EI levels across the domains. Wellbeing recorded the highest score (3.7 ± 0.38), reflecting positive emotions and a sense of relative satisfaction and fulfillment among participants. It was followed by sociability (3.3 ± 0.42), which demonstrated a moderate level of comfort in social situations and personal connections. In contrast, self-control (3.2 ± 0.37) and emotionality (3.1 ± 0.41) scores remained comparatively lower. The global score of TEIQue (3.3 ± 0.33) presents a moderate EI assessment worldwide, which is aligned with the patterns of individual domains during this study.

**Table 2:** Mean scores (mean ± SD) of the TEIQue domains and global score among the study participants.

Domains	Mean ± SD
Wellbeing	3.7 ± 0.38
Self-control	3.2 ± 0.37
Emotionality	3.1 ± 0.41
Sociability	3.3 ± 0.42
TEIQue global score	3.3 ± 0.33

TEIQue, Trait Emotional Intelligence Questionnaire; SD, standard deviation.

Table 3 presents the mean response scores for perceived EI domains and overall TEIQue among nurses based on their characteristics. Wellbeing (3.7 ± 0.37) scores were significantly higher for female nurses than for their male counterparts, with a medium to large effect size (3.4 ± 0.42, *p* = 0.019, Cohen’s *d* = 0.66). Nevertheless, no significant differences were observed between genders in self-control, emotionality, sociability, and overall EI (TEIQue). Similarly, wellbeing (3.8 ± 0.37, *p* = 0.013, Cohen’s *d* = 0.570) and emotionality (3.2 ± 0.46, *p* = 0.023, Cohen’s *d* = 0.456) scores were significantly higher for non-Saudi nurses than for Saudi Arabian nurses, with a medium effect size. The nurses with <5 years of experience had significantly higher wellbeing (3.8 ± 0.43, *p* = 0.023, η^2^ = 0.095) and overall EI (3.4 ± 0.40, *p* = 0.026, η^2^ = 0.090) scores with a medium effect size. In contrast, the nurses with >10 years of experience presented the lowest scores. The degree-holding (bachelor’s or higher) nurses demonstrated significantly higher overall EI (3.4 ± 0.36, *p* = 0.034, η^2^ = 0.056) scores with a relatively small effect size. Similarly, the nurses serving in Obstetrics and Pediatrics departments depicted notably higher EI levels, particularly in terms of self-control, emotionality, and sociability.

**Table 3:** Comparison of emotional intelligence (EI) domain scores (mean ± SD) among nurses based on their demographic and professional characteristics.

Characteristics	Wellbeing	Self-control	Emotionality	Sociability	TEIQue
	Mean ± SD	Mean ± SD	Mean ± SD	Mean ± SD	Mean ± SD
Sex (*p*-value^*^)	0.019^***^	0.603	0.096	0.677	0.075
Female	3.7 ± 0.37	3.3 ± 0.39	3.1 ± 0.42	3.3 ± 0.42	3.3 ± 0.34
Male	3.4 ± 0.42	3.2 ± 0.37	2.9 ± 0.30	3.2 ± 0.38	3.1 ± 0.22
Nationality (*p*-value^*^)	0.013^***^	0.061	0.023^***^	0.614	0.065
Saudi	3.6 ± 0.38	3.2 ± 0.28	3.0 ± 0.35	3.3 ± 0.75	3.2 ± 0.26
Non-Saudi	3.8 ± 0.37	3.3 ± 0.44	3.2 ± 0.46	3.2 ± 0.49	3.4 ± 0.39
Age categories (*p*-value^**^)	0.875	0.740	0.849	0.421	0.797
<30 years	3.6 ± 0.39	3.2 ± 0.39	3.1 ± 0.37	3.3 ± 0.46	3.3 ± 0.28
30–<40 years	3.7 ± 0.36	3.3 ± 0.37	3.1 ± 0.44	3.3 ± 0.40	3.3 ± 0.36
>50 years	3.7 ± 0.63	3.2 ± 0.29	3.0 ± 0.23	3.0 ± 0.38	3.2 ± 0.26
Educational level (*p*-value^*^)	0.096	0.270	0.081	0.276	0.034^***^
Diploma	3.5 ± 0.34	3.2 ± 0.26	3.0 ± 0.28	3.2 ± 0.35	3.2 ± 0.21
Bachelor and above	3.7 ± 0.39	3.3 ± 0.40	3.2 ± 0.45	3.3 ± 0.44	3.4 ± 0.36
Years of experience in current specialty (*p*-value^**^)	0.023^***^	0.358	0.075	0.541	0.026^***^
<5 years	3.8 ± 0.43	3.3 ± 0.42	3.3 ± 0.50	3.3 ± 0.49	3.4 ± 0.40
5–<10 years	3.7 ± 0.34	3.2 ± 0.34	3.0 ± 0.40	3.3 ± 0.46	3.3 ± 0.31
≥10 years	3.5 ± 0.24	3.2 ± 0.33	3.1 ± 0.41	3.2 ± 0.31	3.2 ± 0.24
Years of experience in current hospital (*p*-value^**^)	0.129	0.220	0.027^***^	0.562	0.043^***^
<5 years	3.8 ± 0.43	3.4 ± 0.43	3.3 ± 0.50	3.3 ± 0.49	3.4 ± 0.41
5–<10 years	3.7 ± 0.34	3.2 ± 0.35	3.0 ± 0.40	3.3 ± 0.46	3.3 ± 0.31
≥10 years	3.6 ± 0.37	3.2 ± 0.33	3.0 ± 0.30	3.3 ± 0.31	3.2 ± 0.25
Department (*p*-value^**^)	0.118	0.011^***^	0.018^***^	0.003^***^	0.002^***^
Medicine	3.7 ± 0.36	3.3 ± 0.36	3.1 ± 0.49	3.2 ± 0.47	3.3 ± 0.36
Surgery	3.6 ± 0.45	3.2 ± 0.33	3.0 ± 0.40	3.3 ± 0.38	3.3 ± 0.34
Obstetrics	3.8 ± 0.38	3.6 ± 0.47	3.4 ± 0.41	3.7 ± 0.38	3.6 ± 0.35
Pediatrics	3.8 ± 0.26	3.3 ± 0.25	3.3 ± 0.36	3.3 ± 0.36	3.4 ± 0.21
OPD	3.4 ± 0.33	3.1 ± 0.15	2.9 ± 0.22	3.0 ± 0.21	3.1 ± 0.11
Endoscopy	3.6 ± 0.36	3.0 ± 0.33	3.0 ± 0.16	3.1 ± 0.25	3.1 ± 0.15

* Independent sample *t*-test.

** ANOVA test.

*** Statistically significant.

The regression analysis predicted EI among nurses based on specific work-related factors (Table 4). The significant intercept (B = 3.159, *p* < 0.001) represents the baseline EI level. It confirmed consistency across the sample population, whereas other variables presented non-significant associations. Years of expertise in the field did not notably impact the EI. Similarly, the education level (diploma vs. bachelor’s/postgraduate) did not significantly affect the EI during this study. However, working in the Obstetrics department/unit remained a statistically significant predictor (B = 0.376, *p* = 0.001), which suggested higher EI levels among nurses of the Obstetrics as compared to other departments (B = −0.108, *p* = 0.167). *R*^2^-value (0.131) depicted that 13.1% of changes in EI level can be attributed to the mentioned factors in addition to other factors, which significantly impacted the EI of nurses. Factors such as working hours or organizational policies were not examined due to the study’s focus on TEIQue-SF and sociodemographic/professional variables.

**Table 4:** Regression analysis for emotional intelligence prediction in nurses.

Parameter	B	SD	95% Wald CI		Hypothesis test		
			Lower	Upper	Wald Chi-Square	df	*p*-value
(Intercept)	3.159	0.0977	2.968	3.351	1046.589	1	0.000
Years of experience in the current specialty							
<5 years	0.079	0.1726	−0.259	0.417	0.210	1	0.647
5–<10 years	0.005	0.0875	−0.166	0.177	0.003	1	0.953
10 + years	0a	.	.	.	.	.	.
Department/unit							
General medicine	0.106	0.1068	−0.103	0.315	0.982	1	0.322
Surgery	0.024	0.1119	−0.195	0.244	0.048	1	0.827
Obstetrics	0.376	0.1165	0.147	0.604	10.406	1	0.001^*^
Pediatrics	0.251	0.1302	−0.004	0.506	3.725	1	0.054
OPD	−0.037	0.1292	−0.290	0.216	0.081	1	0.776
Endoscopy	0a	.	.	.	.	.	.
Qualifications							
Diploma	−0.108	0.0783	−0.262	0.045	1.907	1	0.167
Bachelor/postgraduate	0a	.	.	.	.	.	.
(Scale)	.076b	0.0121	0.056	0.104			

*R*^2^ = 0.131.

* Statistically significant.

## 4 Discussion

The study examined EI levels and their influencing factors among nurses from a general hospital in Jeddah, Saudi Arabia. The results revealed moderate EI levels among participating nurses. Contrarily, studies in the United Kingdom have often reported higher EI scores among nurses (10). The contradiction could be due to the more reserved emotional trait in Saudi society. It could be potentially shaped by the cultural norms of Saudi Arabia, emphasizing communal harmony rather than the expression of personal emotions. This background might have enhanced a more reserved emotional profile among participating nurses (18). On the other hand, higher EI scores among Western nurses are associated with individualistic characteristics (8). Differential nursing education could also have contributed to the fact that Western nursing programs explicitly incorporate EI training, whereas technical skills have been the priority in Saudi curricula (19).

The findings highlight a positive association between nurses’ wellbeing and years of experience in the same profession, with less experienced nurses (<5 years) showing higher wellbeing scores than those with ≥10 years. This finding may reflect newer nurses’ higher enthusiasm, recent training, and openness to learn, which facilitates readiness to adapt to the work environment, increasing sense of wellbeing, whereas experienced nurses face emotional fatigue due to increased responsibilities (e.g., leadership roles and complex cases), leading to lower resilience in these challenging healthcare roles (20, 21). Sociability scores indicated moderate comfort in social interactions and relationship building, which is essential for communication and teamwork in healthcare settings and for patients’ wellbeing. However, it could be further improved to enhance teamwork and achieve positive patient outcomes (22, 23).

The data revealed gender disparities as well. Female nurses demonstrated significantly higher wellbeing scores than male nurses. These findings were aligned with a previous study that attributed this difference to social and psychological factors (24). Emotional expression and wellbeing are significantly influenced by gender roles in Saudi Arabian society. Women are taught to prioritize empathy and nurturing, which are crucial in caregiving jobs (25). Contrarily, Western studies attributed women’s elevated EI and wellbeing to flexible gender roles and socialization that enhance emotional skills (8). The difference between female nurses’ and male nurses’ mean scores, despite a modest effect size, indicates that female nurses may contribute unique emotional strengths, teamwork, and patient interaction. Further research is needed to explore the practical impacts within Saudi Arabia’s healthcare context.

Ethnicity also significantly impacted nurses’ wellbeing. Non-Saudi nurses demonstrated significantly higher wellbeing scores as compared to Saudi nurses. It could be attributed to better financial incentives and professional growth opportunities for expatriate nurses as they are recruited internationally at attractive salary packages. These benefits could promote their sense of wellbeing (26). Similarly, expatriate nurses demonstrated significantly higher emotionality scores. The tight-knit support groups of expatriate nurses might have contributed to this phenomenon. These groups provide shared cultural experiences and emotional support for enhanced emotionality (27). Moreover, expatriate nurses adapting to new environments should develop strong emotional resilience to address professional challenges away from their countries. This resilience helps in developing a positive emotional state for better stress management and sustained wellbeing (28).

The degree-holding nurses (bachelor’s or higher) exhibited higher EI levels than those with lower education. It suggests their improved empathy, self-awareness, and relationship management skills. Advanced education improves clinical capabilities and facilitates the development of empathy and emotional resilience (29). Higher EI scores of highly qualified nurses reflect their stress-management training and advanced patient interaction skills (30). Notably, EI levels were significantly higher among nurses from the Obstetrics department than their colleagues in other departments. It reveals their distinct interpersonal capabilities that are necessary for this particular field. Obstetrics, involving maternal and neonatal care, often requires stronger stress management, empathy, and patient-centered communication during emotionally charged situations. Nurses’ assistance to patients and families in these situations helps in developing higher EI levels (31).

The moderate EI levels (3.3 ± 0.33) observed in this study, particularly lower scores for self-control (3.2 ± 0.37) and emotionality (3.1 ± 0.41), could reflect the emotional demands of nursing roles in a high-pressure hospital setting, as influenced by measured factors such as experience, gender, nationality, department, and education [1]. In our view, the lower self-control and emotionality scores suggest that nurses face challenges in emotional regulation, potentially due to the intensity of patient interactions in units such as surgery or endoscopy, where EI demands are high [2]. Although factors such as workload or organizational policies may also influence EI, these were beyond the study’s scope but warrant future investigation [3]. Enhancing EI through targeted training could support nurses’ emotional skills, aligning with Saudi Arabia’s Vision 2030 goal of improving patient-centered care [4].

The moderate level of EI observed in nurses in the current study, particularly in self-control and emotionality, could reflect emotional needs in nursing practice under a high-pressure hospital setting, as influenced by the measured factors such as gender, nationality, years of experience in the same profession, and education. In our view, these lower scores suggest that nurses face substantial challenges in emotional regulation, potentially due to workload and demanding patient interactions in units such as Surgery or Endoscopy, where EI is critical (32). Although factors such as workload and organizational policies may influence EI, these were beyond the study’s scope but warrant further investigation. In addition, further studies are necessary to empirically investigate these dynamics. The application of established methods, such as the Maslach Burnout Inventory, could help in understanding their influence within the Middle Eastern healthcare system to deal with the modern challenges of healthcare workers.

## 5 Implications of the study

The current study on nurses’ EI at a general hospital in Jeddah signifies its implications for nursing practice, education, and future investigations within the Saudi healthcare setting. The participating nurses exhibited moderate emotional competence, which necessitated improvement for enhanced clinical outcomes. Hospitals should arrange EI workshops emphasizing stress management and empathy, particularly for male nurses with lower EI. Future studies should involve longitudinal methodology to assess EI progression over time. Moreover, qualitative investigations are necessary to understand factors associated with wellbeing in female nurses while considering cultural influences such as gender roles. The study offers practical approaches to improve nursing practices and their wellbeing in Saudi Arabia. However, the extension of this investigation to rural areas could aid in assessing its applicability.

## 6 Conclusion

The study revealed moderate EI levels among nursing participants, with wellbeing being the highest component and self-control and emotionality being the lowest component, which were significantly influenced by gender, nationality, and years of experience in the same profession. Female nurses presented significantly better wellbeing scores, whereas wellbeing and emotionality scores were high in non-Saudi nurses. Interestingly, less experienced nurses scored an overall higher EI. Furthermore, nurses from the Obstetrics unit had the highest TEIQue global score. The strengths of the study include the application of a validated instrument (TEIQue-SF), and the inclusion of diverse demographic and professional traits enabled a deeper insight into EI-affecting factors in nurses. However, limitations involve the small sample and single-hospital setting, limiting the generalizability of the results. Besides, the inherited limitation of cross-sectional design indicates that a causal relationship between variables cannot be established.

### 6.1 Recommendations

Based on the findings of moderate EI among nurses with lower self-control and emotionality, we recommend training programs focusing on emotional regulation and empathy skills, tailored for experienced nurses. Such training programs align with EI theory, aiming at patient-centered high-quality care, crucial to Saudi Arabia’s Vision 2030. Future studies are needed with multicenter incorporation and including other potential factors influencing EI in nurses, and to investigate the impact of EI on care outcomes.

## Data Availability

The raw data supporting the conclusions of this article will be made available by the authors, without undue reservation.
