# Correction: Emotional intelligence among nurses in a general hospital setting in Jeddah, Saudi Arabia

**DOI:** 10.3389/fmed.2025.1741878

**Published:** 2025-11-21

**Authors:** Ibrahim Yahya Alhakami, Omar Ghazi Baker

**Affiliations:** 1 College of Nursing, King Saud University, Riyadh, Saudi Arabia; 2 Department of Community Psychiatric and Mental Nursing, College of Nursing, King Saud University, Riyadh, Saudi Arabia

**Keywords:** emotional intelligence, level, influencing factors, nursing, general hospitals, Saudi Arabia

Affiliation 1 “College of Nursing, King Saud University, Riyadh, Saudi Arabia” was erroneously given as “Aleith General Hospital, First Health Cluster, Ministry of Health, Jeddah, Saudi Arabia”.

The original version of this article has been updated.

